# Mechanical characterization of porcine ureter for the evaluation of tissue-engineering applications

**DOI:** 10.3389/fbioe.2024.1412136

**Published:** 2024-06-17

**Authors:** Martina Casarin, Ilaria Toniolo, Martina Todesco, Emanuele Luigi Carniel, Laura Astolfi, Alessandro Morlacco, Fabrizio Dal Moro

**Affiliations:** ^1^ Department of Surgery, Oncology and Gastroenterology, University of Padua, Padova, Italy; ^2^ Department of Industrial Engineering, University of Padua, Padova, Italy; ^3^ Department of Civil, Environmental and Architectural Engineering, University of Padua, Padova, Italy; ^4^ Bioacoustics Research Laboratory, Department of Neuroscience DNS, University of Padova, Padova, Italy

**Keywords:** ureter, tissue-engineering, biomaterials, biomechanics, membrane flexion tests, ring tests, computational model

## Abstract

**Introduction:** Clinics increasingly require readily deployable tubular substitutes to restore the functionality of structures like ureters and blood vessels. Despite extensive exploration of various materials, both synthetic and biological, the optimal solution remains elusive. Drawing on abundant literature experiences, there is a pressing demand for a substitute that not only emulates native tissue by providing requisite signals and growth factors but also exhibits appropriate mechanical resilience and behaviour.

**Methods:** This study aims to assess the potential of porcine ureters by characterizing their biomechanical properties in their native configuration through ring and membrane flexion tests. In order to assess the tissue morphology before and after mechanical tests and the eventual alteration of tissue microstructure that would be inserted in material constitutive description, histological staining was performed on samples. Corresponding computational analyses were performed to mimic the experimental campaign to identify the constitutive material parameters.

**Results:** The absence of any damages to muscle and collagen fibres, which only compacted after mechanical tests, was demonstrated. The experimental tests (ring and membrane flexion tests) showed non-linearity for material and geometry and the viscoelastic behaviour of the native porcine ureter. Computational models were descriptive of the mechanical behaviour ureteral tissue, and the material model feasible.

**Discussion:** This analysis will be useful for future comparison with decellularized tissue for the evaluation of the aggression of cell removal and its effect on microstructure. The computational model could lay the basis for a reliable tool for the prediction of solicitation in the case of tubular substitutions in subsequent simulations.

## 1 Introduction

Several conditions can impair the ureteral function, including congenital malformations, iatrogenic injuries (e.g., due to abdominal and pelvic surgery) and acquired conditions (e.g., urinary calculi, retroperitoneal fibrosis, idiopathic stenosis, trauma, fistula or obstruction of the urine, infections and cancer) (Kloskowski et al., 2013; Xu et al., 2020). Surgery is required in most of these cases to restore ureter function and prevent further kidney damage. These approaches include ureteroureterostomy with end-to-end anastomoses of the same ureter, re-anastomosis with the renal pelvis or bladder, interposition of autologous bowel segments, or placement of indwelling stent dilation when other solutions are not feasible. However, these procedures have various limitations: the first can only be applied to short pathologic ureter segments, the second is limited by the position of the ureteral loss, intestinal substitution cannot fully restore ureter functionality and is often associated with intestinal complications (e.g., diarrhea, infection, nausea, and gastrointestinal bleeding), and finally, the indwelling stent is hindered by the need for stent replacement, migration, infections and obstructions (Knight et al., 2013; Casarin et al., 2021; Geavlete et al., 2021).

Thus, clinicians are experiencing a growing need for readily available substitutes (not only ureters, but also for blood vessels, and bile ducts) that can replace autologous or synthetic grafts. Therefore, while synthetic substitutes are widely accessible, their primary drawbacks include heightened infection risks and inflammatory reactions, as well as limited capacity to stimulate cell growth (Kloskowski et al., 2013; de Jonge et al., 2015; Janke et al., 2019). On the contrary, the use of autologous tissues is limited due to their restricted availability and unreliable biological behaviour in terms of vascularity and viability (Knight et al., 2013). Consequently, tissue engineering proposes the use of xenogeneic tissues, appropriately treated to prevent or mitigate potential immunological responses upon implantation. This approach offers biological scaffolds that facilitate tissue regeneration by attracting cells and stimulating their differentiation (Davis et al., 2018).

In pursuit of this goal, ureters have emerged as a potential alternative for ureteral substitution (Hiroshi et al., 2006; Koch et al., 2015; Wang et al., 2021), as well as other types of conduits, including blood vessels (Matsuura et al., 2004; Murase et al., 2006; Narita et al., 2008; Chemla and Morsy, 2009) and bile ducts (Cheng et al., 2016), due to their appropriate length, diameter, and lack of luminal valves (Koch et al., 2015). The short-term goal of the present study consists of investigating the mechanical feasibility of porcine ureters to assess their eventual application as a valid biological tissue substitute.

Until now, mechanical tests on ureters have been performed in humans (Rassoli et al., 2014; Shilo et al., 2014; Sokolis, 2019) and different animal models (Yin and Fung, 1971; Weiss et al., 1972), but only few data were reported for porcine model. Uniaxial tests were performed along the circumferential and longitudinal directions to evaluate the tensile strength and longitudinal direction was stiffer than circumferential one (Van Mastrigt et al., 1981).

The purpose of this preliminary work was to test the mechanical response of native porcine ureteral tissue by performing a mechanical characterization, at both tissue and substructural levels, using ring tensile and membrane flexion tests, enriching the poor mechanical and morphological evaluations performed by others on ureters (O’Meara et al., 2024). The ring tensile set-up permitted the avoidance of invasive sample cutting, as performed in the standard uniaxial tensile test, preserving the circular nature of the ureter, which functions as a conduit. However, the test solicited the sample only in a single direction, hence the membrane flexion test was performed to also analyse the contribution of different fibre orientations in a sub-structural configuration. Computational analyses were performed aimed at reproducing the experimental tests reported above to propose and validate a material formulation and material constitutive parameters of porcine ureters (Salmaso et al., 2020; Toniolo et al., 2022).

## 2 Materials and methods

### 2.1 Samples preparation

Tissue samples from slaughtered pigs were used in this study. The procedures followed by the slaughterhouse were authorized by the relevant animal welfare legal authorities (Food and Consumer Product Safety Authority) and were in accordance with EC regulations 1099/2009 regarding the health and protection of animals at the time of slaughter. They were also overseen by the Italian government.

Within 3 h of the porcine urinary system explant provided by a local slaughterhouse, ureters were isolated in the laboratory by cannulating them with a 2 mm diameter catheter and removing the external adipose and fibrous tissues with surgical scissors. The ureters were then washed in saline solution and cryopreserved in a solution consisting of 10% Fetal Bovine Serum (FBS) and 10% dimethyl sulfoxide (DMSO) diluted in Dulbecco Modified Eagle Serum high glucose (DMEM high glucose) at −80°C (Casarin et al., 2023b). Before proceeding to laboratory investigations, the tissues were thawed in a water bath at 37°C and then soaked in saline solution, which was repeatedly refreshed until it remained completely transparent.

### 2.2 Paraffin-embedding and histological evaluations of the ureter samples

All ureter samples, including controls (unstressed native samples) and tested samples (native samples subjected to the mechanical tests), were fixed in Shandon Glyo-fixx (Thermo Fisher Scientific) for 6 days at 4°C. Then, they were washed for 5 min in tap water and soaked overnight in 70% ethanol at 4°C before proceeding with the paraffin embedding of the samples. This process involved dehydratation followed by diaphanization. Paraffin-embedded samples were cut using the semi-automatic microtome CUT 5062 (SLEE medical GmbH, Mainz, Germany) into sections with a thickness of 4 µm. After rehydration, the sections were stained with Mayer Hematoxylin (Leica Microsystems, Milan Italy) and 1% aqueous Eosin (Leica Microsystem) to evaluate the overall tissue structure. Masson Trichrome (Bio-Optica, Milan Italy) was used to highlight collagen fibres with aniline blue and muscle fibres in red. Additionally, 0.1% Picrosirius Red solution (Direct Red 80, Merck KGaA) in a picric acid-saturated aqueous solution (Merck KGaA) was used to visualize different collagen fibres. Histological images of Hematoxylin and Eosin (HE) and Masson Trichrome (MT) were acquired using the light microscope ECLIPSE 50i (Nikon) with Nis-Elements 3.0 Image Analysis System software (Nikon, Amsterdam, Netherlands). Images of the Picrosirius Red (PR) staining were captured using a polarized light microscope Leica DMRE (Leica, Wetzlar, Germany).

### 2.3 Mechanical tests

**FIGURE 1:**
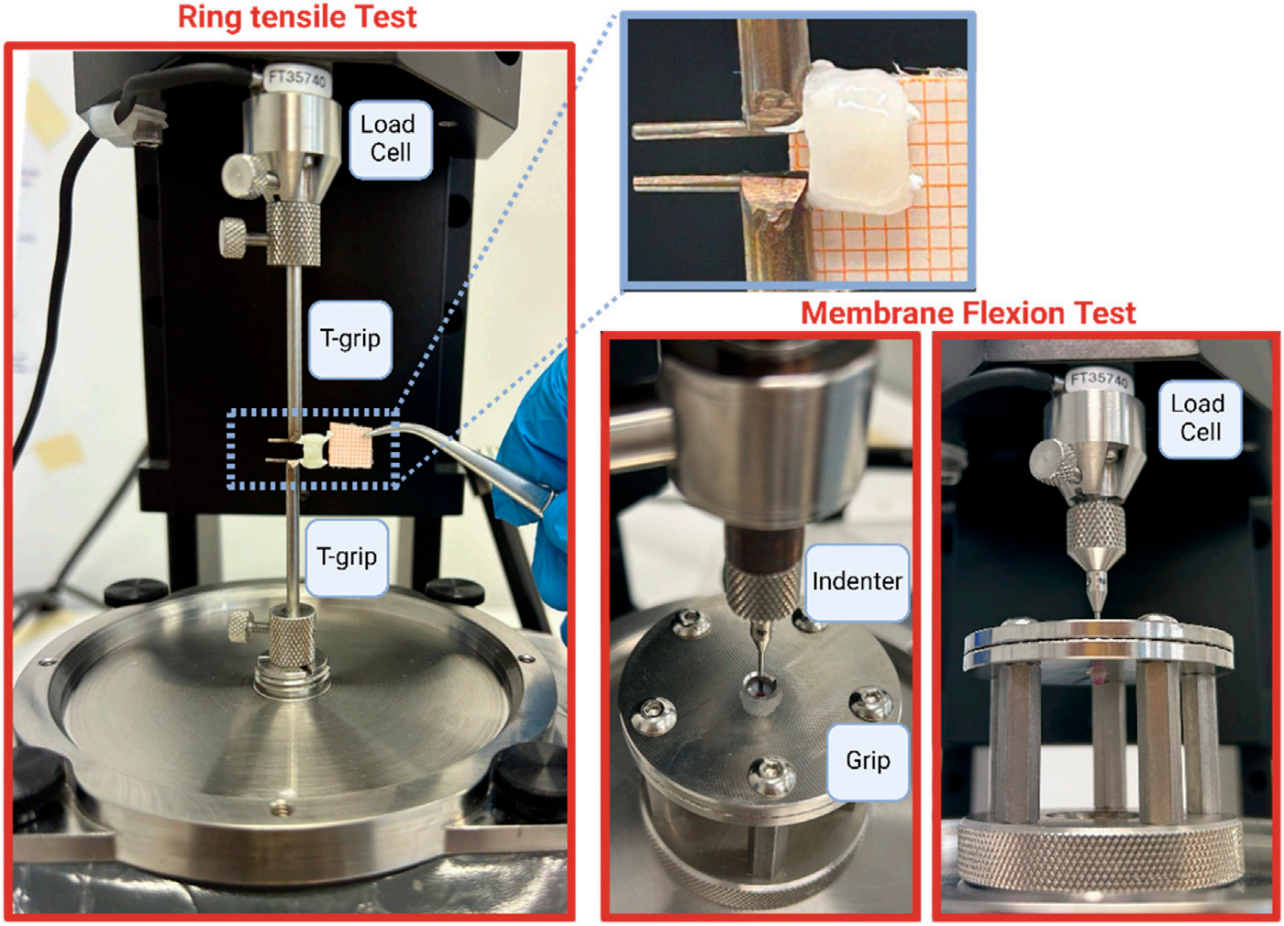
Experimental set-up for ring and membrane flexion tests.

Ring tensile and membrane flexion tests were conducted using the Biomomentum testing machine (Model Mach-1 v500css, ©Biomomentum Inc., Laval, QC, Canada), which featured a multi-axial load cell with a 70 N capacity and 0.0035 N resolution. For ring tensile tests, the machine was outfitted with two T-grips to secure the ureteral rings, while for membrane flexion tests, a spherical 2 mm-diameter indenter was directly affixed to the load cell. Additionally, two spherical plates with 5 mm-diameter holes were utilized for membrane flexion tests. In this configuration, ureteral samples were positioned between the plates, with grooved sides facing inward (Figure 1).

The preparation of samples for the ring test involved selecting approximately a 5 mm-length tubular ureter portion, while for the membrane flexion test, it consisted of selecting approximately a 7 mm-length tubular ureter portion and opening it by inserting scissors into the lumen. The internal surface was placed in contact with the spherical indenter.

All components utilized were crafted from stainless steel 316 L material. Throughout preparation and testing, samples were consistently moistened with saline solution at room temperature. Sample preconditioning was conducted for both test types, involving five loading-unloading cycles with a strain of 1% and a displacement of 0.1 mm. The ring and membrane flexion tests were performed at a strain rate of 20%/s and 0.5 mm/s for, respectively, and a number of five steps were conducted (Table 1). Each step was followed by a rest period of 300 s. The objective of both tests was to analyse the time-dependent behaviour of ureteral tissue, specifically through stress and force relaxation tests. Detailed methodologies are provided in Table 1. Following the collection of equilibrium points, the equilibrium behaviour was fitted with an exponential function (Eq. 1), while relaxation phenomena were modelled using a 3-branch Prony model (Eq. 2):

$$y=a*\left[\exp\right.\left(b*x\right)\left.-1\right]$$ (1)

$$y=1-\gamma_{1}*\left(1-\exp\left(-\frac{x}{\tau_{1}}\right)\right)-\gamma_{2}*\left(1-\exp\left(-\frac{x}{\tau_{2}}\right)\right)-\gamma_{3}*\left(1-\exp\left(-\frac{x}{\tau_{3}}\right)\right)$$ (2)

**TABLE 1:** Experimental protocols.

Test typology	Samples	Number of steps	Strain [%] or displacement [mm]	Relaxation time (s)	Acquisition frequency (Hz)
Ring tensile Test	7 animals–13 samples	8	20%	300	100
Membrane Flexion test	8 animals–13 samples	5	1 or 2 mm	300	100

Hence, the equilibrium behaviour and the stress and the force relaxation phenomenon, for the ring and membrane tests respectively, were the main outputs used to identify the mechanical response of the ureteral tissue.

### 2.4 Computational activities

**FIGURE 2:**
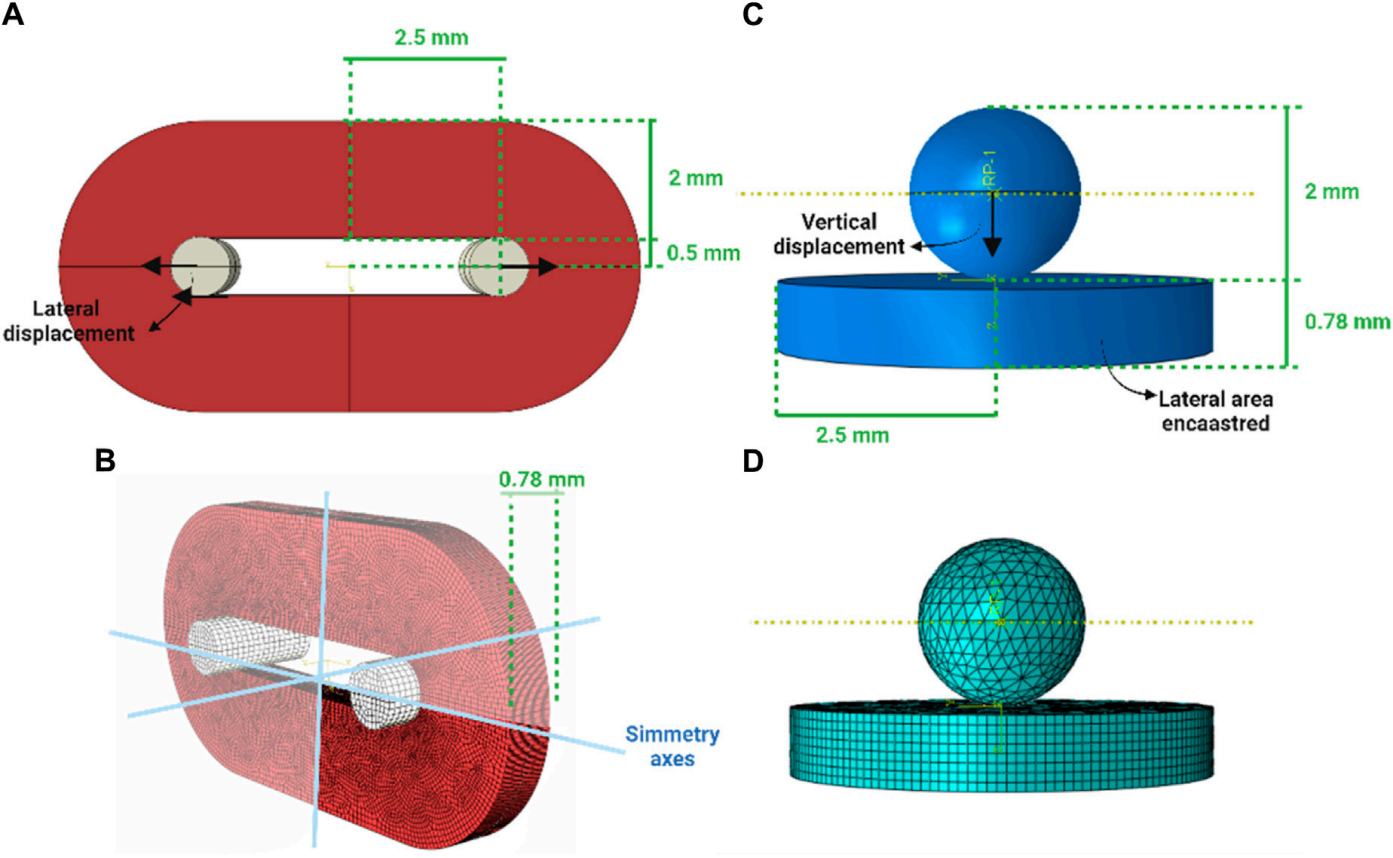
Virtual solid models of the ring and membrane flexion test **(A,C)** and the discretisation to the finite elements **(B,D)**, respectively.

To reproduce the ring tensile test, symmetries were exploited and only an octave of the entire configuration was generated. The model accounted for 94820 nodes and 86624 linear hexahedral elements of type C3D8R. The portion related to the T grip was simplified as a rigid body and an elastic material (Young’s modulus of 10 GPa and Poisson coefficient of 0.3) was assigned, and a lateral displacement of 8 mm associated to a 160% of engineering strain was imposed to it (Figures 2A, B). To reproduce the membrane flexion test, a disk of 5 mm of diameter and 0.78 mm of thickness to simulate the biological sample and a 2-mm-diameter sphere were generated. The model accounted for 54441 nodes and 54085 linear hexahedral elements of type C3D8R (for the disk) and C3D4 (of the sphere) (See Appendix 1). The sphere was simplified as a rigid body and an elastic material (Young’s modulus of 10 GPa and Poisson coefficient of 0.3) was assigned, then a vertical displacement of 5 mm was imposed. The lateral area of the disk was fixed (encastre) (Figures 2C, D). To the disk, the same hyperelastic material was imposed. The contact imposed between the surface of the spherical indenter and the biological tissue was Hard-contact type and frictionless, while in the ring test model a tie condition was imposed between the T grip and the ureter.

The simulation step time was 10 s and were performed on Abaqus 2023 (Dassault System).

To the ureter an hyperelastic first degree Ogden material model $U=-p*\left(J-1\right)+\frac{2\mu }{\alpha^{2\;}}\left(\lambda_{1}^{\alpha }+\lambda_{2}^{\alpha }+\lambda_{3}^{\alpha }-3\right)$ was assigned, where *U* is the strain energy potential expressed in terms of the principal stretches $\lambda_{i}$ , J is the deformation Jacobian and p is the hydrostatic pressure, which is introduced to enforce tissue incompressibility condition. The tissue was considered incompressible. The constitutive material parameters were extracted from the fitting of the median curve behaviour of the experimental results obtained followed ring tensile test using the tool present in Abaqus 2023 called material evaluator. The parameters that best identified the curve were µ = 1.07 kPa and α = 6.60 [-]. The parameters were then used in the ring and membrane flexion model to replicate the experimental campaign and validate them.

As for the experimental activities, the equilibrium behaviour was the main output used to identify the mechanical response of the ureteral tissue, since the simulation did not include visco-elasticity. The forces were acquired from the reference point associated to the rigid bodies (the T-grip and the spherical indenter) and, in case of ring test, transformed in the corresponding stress by dividing for the transversal section.

## 3 Results

**FIGURE 3:**
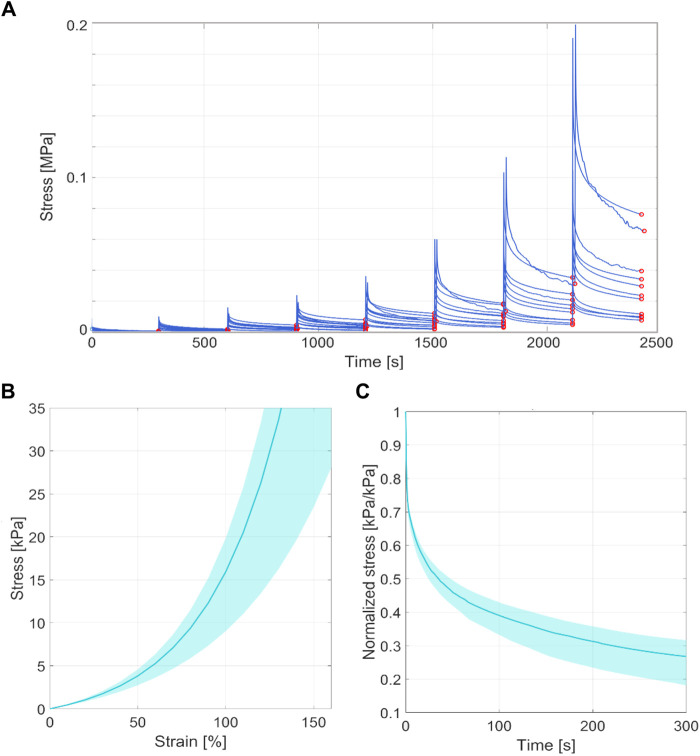
**(A)** Coarse data of ring tensile test of all the tested samples in terms of stress-time. The red circles identified the equilibrium point at the end of the stress relaxation. **(B)** Statistical band (from 25% to 75% of confidential interval) of the equilibrium behaviour and median curve. **(C)** Statistical band (from 25% to 75% of confidential interval) of the relaxation behaviour and median curve.

**FIGURE 4:**
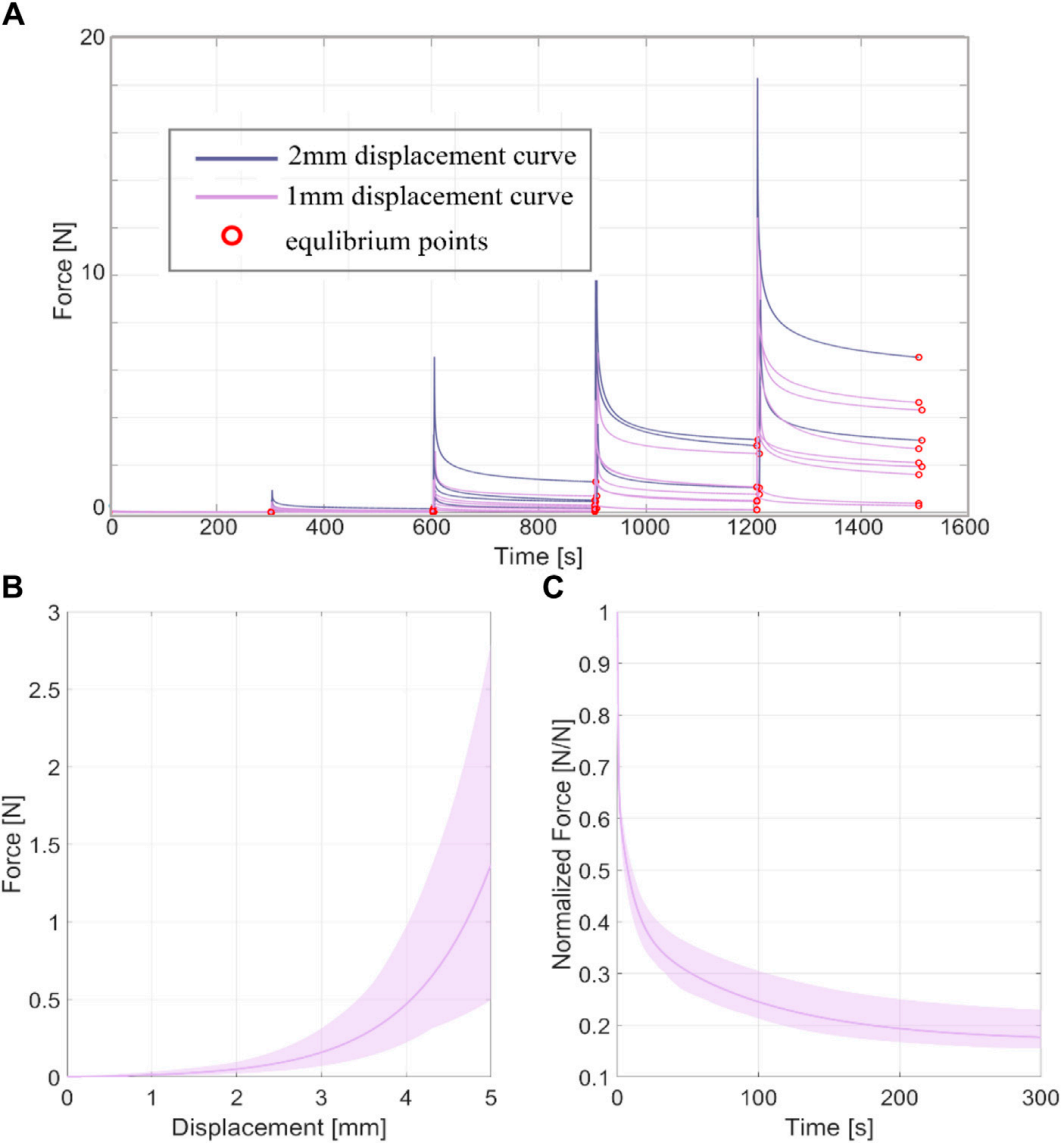
**(A)** coarse data of membrane flexion test of all the tested samples in terms of force-time. The red circles identified the equilibrium point at the end of the force relaxation. **(B)** Statistical band (25%–75% of confidential interval) of the equilibrium behaviour and median curve. **(C)** Statistical band (25%–75% of confidential interval) of the relaxation behaviour and median curve.

The thickness of all the samples was on average 0.78 mm (±0.14 mm), measured using a digital calibre. In Figures 3A, 4A, the raw data related to each sample for the ring and membrane flexion tests, respectively, were proposed. These curves were post-processed to extract the equilibrium point (light red circles), that were the points at the end of each relaxion time to obtain the equilibrium behaviour (Figures 3B, 4B). Each stress or force relaxation (the signal between the peak and the equilibrium point) was considered and normalized by the peak. The relaxation behaviour was then depicted in Figures 3C, 4C. The statistical bands, representing the distribution between the 25th and 75th % of the confidential interval, were wide, and the standard deviation increased as the test proceeded. This is a typical feature of biological tissues that are affected by high inter-sample variability (Fontanella et al., 2022). The material parameters related to Eqs 1, 2, which describe the median equilibrium and relaxation behaviours, are reported in Table 2.

**TABLE 2:** Constants of the median curve and standard deviations for ring tensile and membrane flexion tests (Eq. 1, 2).

	*a* *[* *kPa]*	*b [-]*	*τ*1 *[* *s]*	*τ*_*2*_ *[* *s]*	*τ*_*3*_ *[* *s]*	*γ*_*1*_[*-*]	*γ*_*2*_[*-*]	*γ*_*3*_[*-*]
Ring tensile Test	*0.0018 (±0.0025)*	*0.0231 (±0.008)*	*0.7618 (±0.23)*	*17.17 (±5.064)*	*177.1 (±33.38)*	*0.2617 (±0.03)*	*0.2104 (±0.04)*	*0.3194 (±0.25)*
Membrane Flexion test	*0.0068 (±0.035)*	*1.0606 (±0.33)*	*0.5159 (±7.57)*	*9.374 (±3.48)*	*89.12 (±85.81)*	*0.3414 (±0.04)*	*0.2548 (±0.07)*	*0.2352 (±0.05)*

**FIGURE 5:**
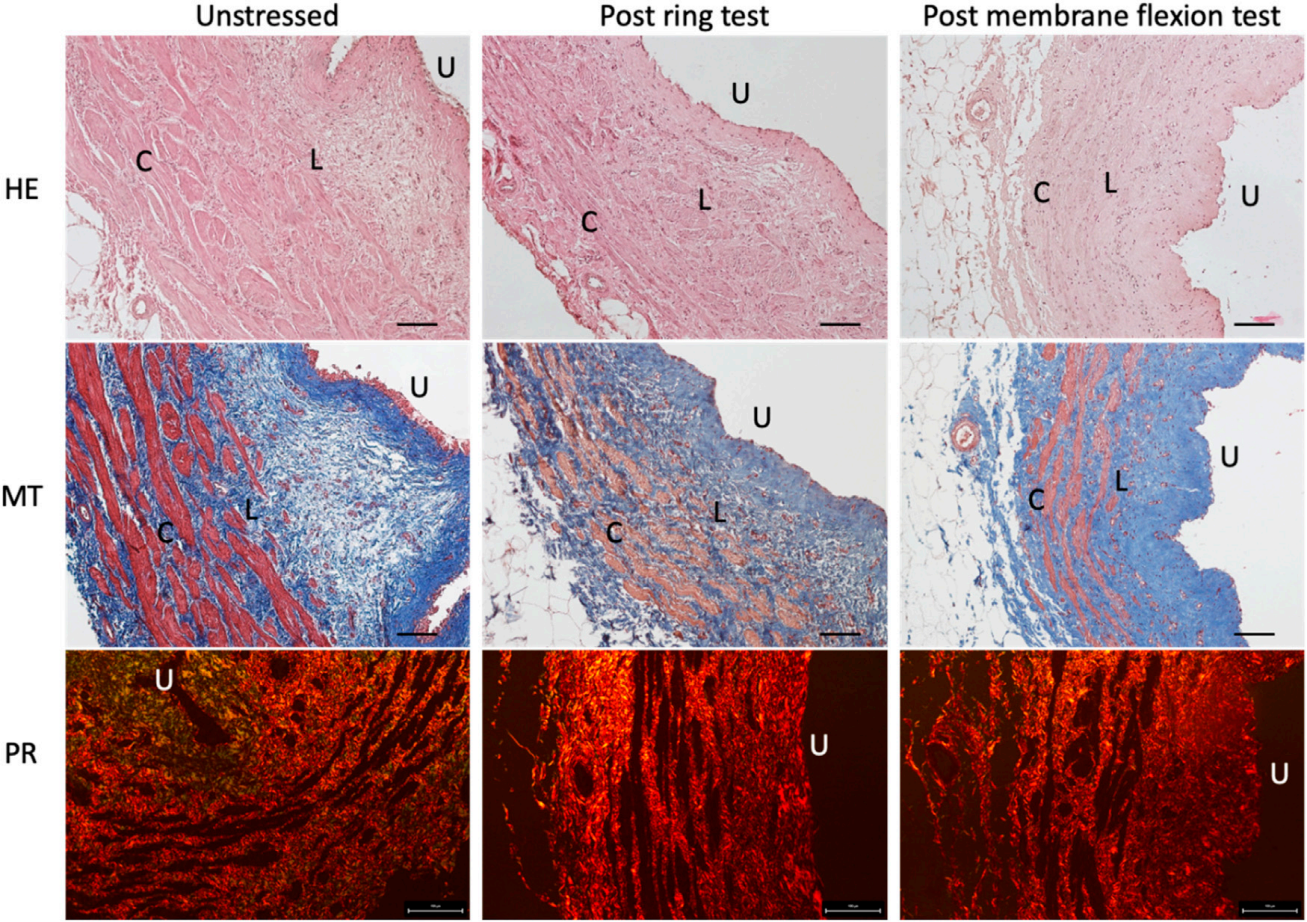
Histological staining of the native ureters before (first column) and after ring test (second column) and membrane flexion test (third column). In the first row Haematoxylin and Eosin (HE) staining is reported to show in blue the nuclei and in pink the cytoplasm; in the second row Masson’s Trichrome (MT) is showed to highlight in blue the collagen fibres and in red the muscle fibres; finally, in the third row Picrosirius Red (PR) shows in red the collagen fibres. Scale bars: 100 µm. U = urothelium, L = muscle fibres in longitudinal direction, C = muscle fibres in circumferential direction.

The microscopic composition and structure of the ureter were evaluated using Haematoxylin and Eosin staining (HE), Masson’s Trichrome staining (MT) and Picrosirius Red staining (PR) (Figure 5). The native ureter is a muscular conduit composed of two smooth muscle layers: the external one is organized in the circumferential direction C), while the internal one in the longitudinal direction L). In the lumen, a transitional epithelium (urothelium) is present U) (Figure 5 HE images). Before and after both mechanical tests (ring and membrane flexion), the ureter structure and morphology are not negatively affected, maintaining its collagen and muscle fibres organization and orientation (Figure 5, MT e PR images). The only differences observed are in terms of an increased compactness of the tissue after the mechanical tests (as expected), with almost no gaps between collagen fibres (seen as the blue colour in Figure 5-MT) and the loss of the most externally exposed layer of urothelium (seen as the red colour in Figure 5-MT, especially on the “U” side).

**FIGURE 6:**
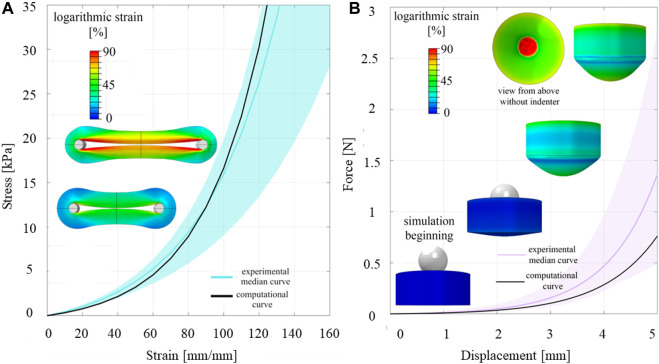
Comparison between experimental and computational results of ring **(A)** and membrane flexion test **(B)**.

In Figure 6, a comparison between experimental and computational results is reported. Computational results (black curves) fall into the corresponding experimental statistical bands, that means that the computational FE models proper described the experimentations in terms of material formulation and boundary conditions. For the ring tensile test, the root means square error amounted to 9 kPa when the median experimental behaviour and the computational curve were considered, while for the membrane flexion tests to 0.18 N. The results were considered acceptable due to the treatment of biological materials with the above-mentioned features.

## 4 Discussion

Ureters are double J-shaped conduits, typically 25–30 cm long, which connect the kidneys to the urinary bladder. The main function of the ureter is to transport urine to the urinary bladder through the peristaltic action of its smooth muscle layers. Histological analyses performed in this study reveals that the ureter is coated with two spirally arranged muscle bundles: the outer layer is circular C), while the inner one is longitudinal L) (Figure 6). These muscle layers are fused by connective tissue and are responsible for the bilateral alternating peristaltic movements, with a velocity of 2–3 cm/s. Below the muscle layers is the mucous membrane and the urothelium, an epithelium underlying the lamina propria, which line the lumen of the ureter (U) (Fröber, 2007).

Recent attention has been directed toward tissue-engineering techniques aimed at providing ready-to-use substitutes for ureteral tissues and other urinary conduits (Casarin et al., 2021; 2022b), as demonstrated in the present study with the evaluation of the mechanical suitability of the porcine ureter. To date, several studies have been conducted on small intestinal submucosa (SIS) (Shalhav et al., 1999; Thomas et al., 2002) and urinary bladder matrix (UBM) (Liu et al., 2009) to be used as urinary conduits. These studies have shown enhanced support for cells growth, proliferation, and differentiation of multi-layered urothelium and smooth muscle cells, while maintaining the connective tissue, which acts as a scaffold to provide mechanical support. Unfortunately, the mechanical support provided is often insufficient for the chosen application, especially in the case of full-circumferential defects (Shalhav et al., 1999). To address this issue and make tissues with limited biomechanical resistance applicable overcoming the mechanical limitations of decellularized SIS (Casarin et al., 2022a), recent studies have proposed the coupling of the biological tissue and a synthetic biomaterial to provide appropriate mechanical support in combination with the biological activity of the tissue (Casarin et al., 2022c). This hybrid scaffold represents a novel type of material proposed for biomedical applications, including urinary and cardiovascular ones (Casarin et al., 2023c; Todesco et al., 2023). Nevertheless, many other types of biomaterials have been investigated for ureteral substitution, such as vein grafts (Wolters et al., 2010; Zhao et al., 2019; Casarin et al., 2023a), collagen (Vardar et al., 2015; De Jonge et al., 2018) and notably animal ureters (Dahms et al., 1997; Shalhav et al., 1999; Osman et al., 2004; Hiroshi et al., 2006; Meng et al., 2015; Xiao et al., 2016; Wang et al., 2021). Ureter tissue has also been investigated for the blood vessel substitution (Ketharanathan and Christie, 1982; Roberts et al., 1989; Clarke et al., 2001; Field, 2003; Matsuura et al., 2004; Darby et al., 2006; Emrecan et al., 2006; Tolva et al., 2007; Derham et al., 2008; Spark et al., 2008) and even for bile duct substitution (Cheng et al., 2016), as it possesses adequate length, diameter, and a strong tissue matrix. However, despite numerous efforts to develop materials with adequate biological and mechanical properties, the authors believe that, whenever possible, the preferred choice should be the same tissue that is to be replaced, as it already possesses the necessary properties of biomechanical strength and promotes appropriate cell growth.

As it is known, animal tissues cannot be directly applied to *in vivo* implantation due to the strong immunological rejection they would cause, stemming from the presence of animal cells and DNA within the matrix. For this reason, previous experiments with the bovine ureters have involved the use of crosslinked ureters to overcome this issue. However, this strategy is not optimal as it can hinder cell adhesion and proliferation, and it offers limited mechanical features. Therefore, different approaches have been investigated to avoid this side effect. Initially, fixatives such as carbodiimide (CDI), genipin (GP) and glutaraldehyde (Koch et al., 2015) were utilized to devitalize the scaffolds. Subsequently, attention has shifted to the possibility of completely decellularizing the scaffold with the aim of repopulating it with the resident cells. Decellularized tissues are expected to promote cells growth and organization while possessing more adequate biomechanical resistance. Furthermore, decellularized tissues demonstrated superior biocompatibility and more suitable mechanical behaviour compared to other proposed solutions.

In the present work, the mechanical behaviour of the porcine native ureter was investigated to evaluate its potential application in clinics for the substitution of tubular tissues such as the ureter and blood vessels, through ring and membrane flexion tests. These tests were not reported in literature before, except for ring tests performed on human ureters to investigate the contributions of single layers (collagen, elastin and muscle fibres) to tissue resistance (Sokolis, 2019). Other studies have reported a mean ultimate tensile strength of 2.31 MPa in the circumferential direction (Rassoli et al., 2014) and 5.02 MPa in the same direction, specifying the distal region (Shilo et al., 2014). The experimental results reported in this work were far from these values, suggesting that the chosen range of analysis would not include damages even though the experimental setups did not consider a physiological loads and conditions that require dynamic configurations, the presence of urine or blood (depending on the type of tubular substitution), and the use of intact ureters. Further comparisons were complex due to different protocols and sample preparations. Indeed, in the present study, the ring test allowed for the examination of intact ring sample uniaxially, avoiding the cutting of the sample as in the traditional traction uniaxial tests and preserving its physiological circular shape. However, to study the contribution of the different fibre orientations at a sub-structural level, membrane flexion tests were performed on planar ureter samples by longitudinally cutting the ring. The mechanical characterisation filled the gap in the scientific literature regarding ureteral mechanical properties, which as expected, exhibited a non-linear, high-strain, and time-dependent response, characterized by high variability, especially evident at the final stages of the tests. In addition to analysing the mechanical tests performed, histological staining was conducted pre and post mechanical tests (both ring and membrane flexion tests) to assess any damage to the ureter tissue caused by the high-strain tests. This evaluation was crucial for incorporating the tissue’s response into the material constitutive formulation utilized in the computational models. The histological evaluation aimed to assess the rearrangement of collagen and muscle fibres (highlighted with MT and PR staining) after the designed mechanical tests, in line with another study performed on the pericardium before and after mechanical tests (Bagno et al., 2018). This approach was essential as the ureter’s mechanical resistance was correlated with the collagen and elastin fibres and the different layers of smooth muscle fibres (Rassoli et al., 2014). Moreover, histological analyses can be further utilized to compare native and decellurized tissue in the future, evaluating whether the decellularization process is excessively aggressive, introducing significant changes in the microstructure, thus jeopardizing the tissue’s mechanical response and its suitability as a tubular substitute.

The microscopic observations did not reveal any significant difference, but only an increase in compactness. No changes in the composition of collagen or smooth muscle fibres were observed. Thus, despite deformation at higher stress levels, the tissue can still be considered safe and useable, with some level of security, according to the authors. However, they advise caution. It is important to note that the ureter is a viscoelastic tissue, and the observed time dependency reinforces the previous statement, even if only at the strain rate used in the experiment. Moreover, the tissue displayed laxity at the conclusion of the test, and minimal elastic return was observed, resulting in permanent deformation. Therefore, it is risky to draw further conclusions about the mechanical behaviour beyond the limits investigated.

**FIGURE 7:**
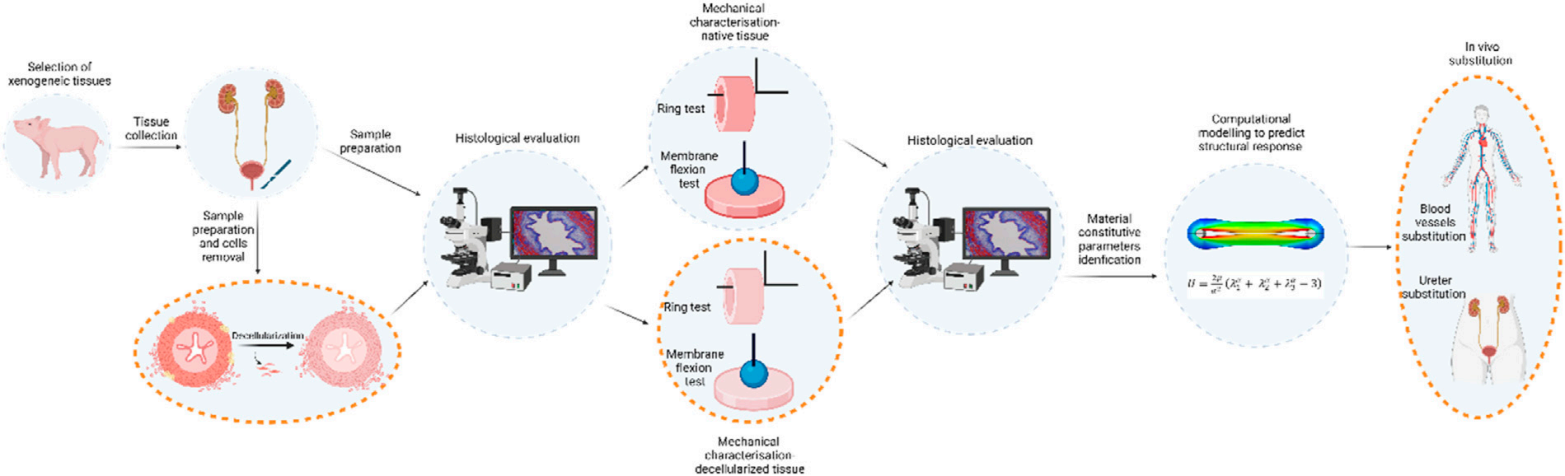
Schematic workflow of the present study and the future steps on mechanically characterisation and morphologically evaluation of the porcine native ureter. Future developments are reported circled with dashed orange line, and they will consist in the ureter decellularization and its mechanical evaluation in order to provide ready-to-use tubular substitutes.

This study was focused on obtaining data describing the mechanical characteristics of native tissues with the aim of producing, through decellularization of porcine ureters, a tubular scaffold that can respond well to mechanical stimuli as an innovative biocompatible matrix suitable for future *in-vivo* implantation. Secondly, but equally important, enriching the computational model with data on the decellularized condition will provide a valid method for predicting the response of tissue-engineered grafts implanted *in vivo* in specific applications (e.g., urological or cardiovascular). The schematic workflow is reported in Figure 7.

Numerical methods can be a powerful tool to deeply analyse different scenarios involving the biological materials (Salmaso et al., 2020; Toniolo et al., 2021), to collect strain and stress distribution (whose analysis is very expensive in experimental setup) and to predict the mechanical response of the tissue. It is worth noting that these evaluations can only be carried out if the material parameters were extracted from experimental evidence (in this case from ring tensile tests) and then validated by other independent tests (in this case the membrane flexion test) (Toniolo et al., 2022).

The present paper included the computational modelling as it is increasingly used to investigate vast surgical scenarios and predict possible controversies. This allows comparisons among innovative techniques and technologies without performing premature clinical trials (Riva et al., 2019; Salmaso et al., 2020; Steer et al., 2021; Hennicke et al., 2022; Mascolini et al., 2023; Toniolo et al., 2024) starting from an experimental campaign of related biological tissues (Carniel et al., 2020; Pettenuzzo et al., 2023). By combining an experimental and computational approach (Toniolo et al., 2022), the constitutive parameters can be obtained after choosing a suitable material model that can describe the main mechanical features and can be validated (intended to be able to accurately predict mechanical behaviour) by comparing the experimental evidence with the model results.

The constitutive material model for computational analyses fell back to one that was incompressible and lacked fibre because of the need to maintain the model the easiest, but descriptive of the experimental data. Moreover, the complexity in cutting the sample around the lumen to experiment different direction was almost impossible due to the extreme fragility in preserving the microstructural arrangement. For these reasons, ring tests was chosen instead of uniaxial tensile tests.

The experimental campaign carried out in this work allowed us to characterize the mechanical behaviour of porcine native ureter as preliminary assessment in view of future surgical applications such as ureter, blood vessel, and bile duct substitutions. The decellularized tissue will need the preservation of the mechanical properties to obtain a scaffold fulfilling the required function and at the same time, to be a valid environment for cell growth. Future research activities will lead to the exploitation of the computational model by simulating blood flow or urine flow, setting the pressure or the flux, and predicting the mechanical response of the ureter, leading to conclusions about its eventual use in the surgical field. The computational models may detect excessive stress or strain that will result in failure.

## 5 Conclusion

The purpose of this preliminary study was achieved by evaluating the mechanical characteristics of the native porcine ureter to assess its potential compatibility for replacing conduits such as ureters and blood vessels. The computational model was created to predict the performance of these grafts after *in vivo* implantation.

The next step is to refine the process of decellularization for porcine ureters to eliminate all cells and DNA that could trigger an immune response. Finally, the necessary mechanical tests described above will be repeated to enhance and validate the predictive value of the computational model.

## Figures and Tables

**FIGURE A1:**
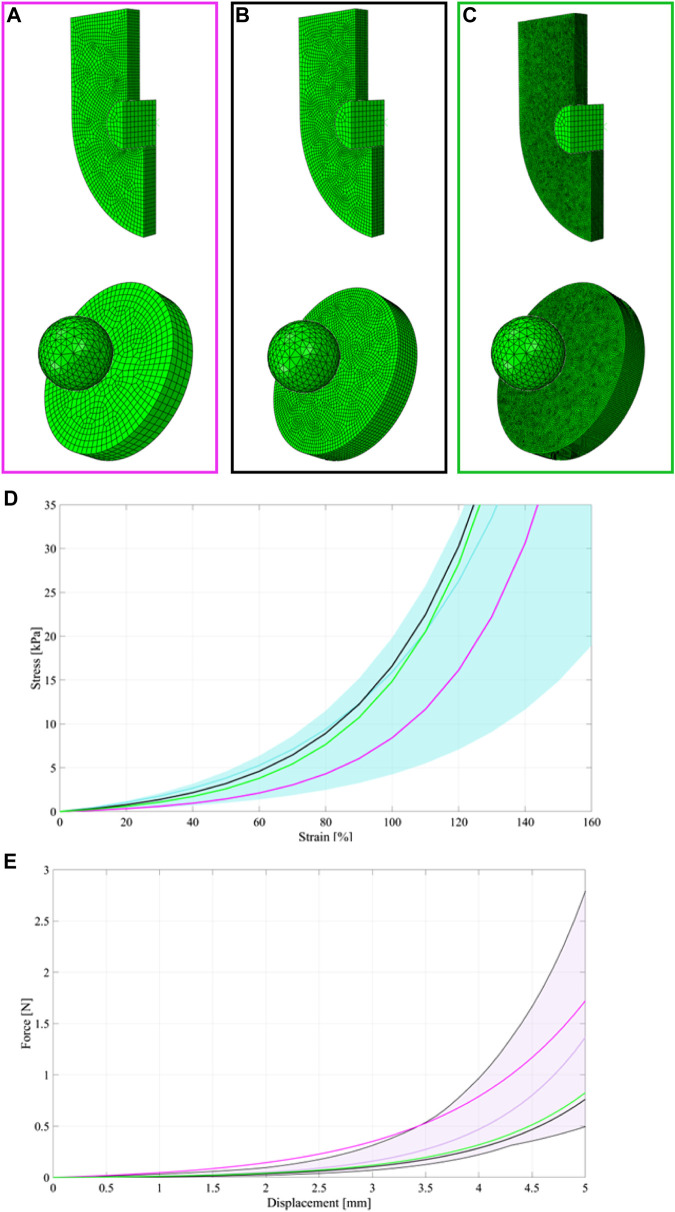
Coarser meshes **(A)**, selected meshes **(B)** and finer meshes **(C)** for the ureter and membrane flexion tests. Experimental statistical bands and median curve for the ring (d – light blue band and light blue median curve) and membrane flexion test (e -light pink band and light pink median curve), respectively. Comparisons between experimental and computational results for the ring **(D)** and membrane flexion test **(E)** for the different finite element discretisation. The black curves were related to the selected discretisations, while the pink and the green curves were related to the coarser and finer meshes, respectively.

## Data Availability

The raw data supporting the conclusion of this article will be made available by the authors, without undue reservation.

## Appendix A

Different finite element discretisations were performed to influence of the size of the element and the computational results. The three considered discretisations for the ring tensile model consisted of 17307, 86624 and 204407 linear hexahedral elements of type C3D8R for the ureter part, while the T-grip discretisation was kept unchanged because it was a rigid body. For the membrane flexion model, the three considered discretisations consisted of 2665, 54085 and 150489 linear hexahedral elements of type C3D8R. The computational meshes and the results were shown and compared with the experimental data (Figure A1A). The curves related to the coarser mesh (pink ones) did not properly interpreter the experimental data in both tests, especially in the membrane flexion test (Figures A1A–E) the curve fall partially out of the confidential interval. The black and the green curves, related to selected and finer mesh respectively were close in both of the cases and for this reason, the authors selected to present in the paper the model that accounted for the lesser number of elements in an optimum of finding the best compromise between computational burden and best fitting. Moreover, the running time of the selected model was one third respect to model with finer mesh.

## References

[B1] Bagno A. Aguiari P. Fiorese M. Iop L. Spina M. Gerosa G. (2018). Native bovine and porcine pericardia respond to load with additive recruitment of collagen fibers. Artif. Organs 42, 540–548. 10.1111/aor.13065 29280157

[B2] Carniel E. L. Albanese A. Fontanella C. G. Pavan P. G. Prevedello L. Salmaso C. (2020). Biomechanics of stomach tissues and structure in patients with obesity. J. Mech. Behav. Biomed. Mater 110, 103883. 10.1016/j.jmbbm.2020.103883 32957190

[B3] Casarin M. Fortunato T. M. Imran S. Todesco M. Sandrin D. Borile G. (2022a). Porcine small intestinal submucosa (SIS) as a suitable scaffold for the creation of a tissue-engineered urinary conduit: decellularization, biomechanical and biocompatibility characterization using new approaches. Int. J. Mol. Sci. 23, 2826. 10.3390/ijms23052826 35269969 PMC8910833

[B4] Casarin M. Fortunato T. M. Imran S. J. Todesco M. Sandrin D. Marchesan M. (2023a). Preliminary *in vitro* assessment of decellularized porcine descending aorta for clinical purposes. J. Funct. Biomater. 14, 141. 10.3390/jfb14030141 36976065 PMC10058365

[B5] Casarin M. Morlacco A. Dal Moro F. (2021). Bladder substitution: the role of tissue engineering and biomaterials. Processes 9, 1643. 10.3390/pr9091643

[B6] Casarin M. Morlacco A. Dal Moro F. (2022b). Tissue engineering and regenerative medicine in pediatric urology: urethral and urinary bladder reconstruction. Int. J. Mol. Sci. 23, 6360. 10.3390/ijms23126360 35742803 PMC9224288

[B7] Casarin M. Todesco M. Fontanella C. G. Morlacco A. Dal Moro F. Bagno A. (2023b). “Cryopreservation effects on biological tissues: histological and mechanical assessments,” in Convegno Nazionale di Bioingegneria.

[B8] Casarin M. Todesco M. Fontanella C. G. Morlacco A. Dal Moro F. Bagno A. (2023c). Hybrid materials for tissue repair and replacement: another frontier in biomaterial exploitation focusing on cardiovascular and urological fields. Processes 11, 2013. 10.3390/pr11072013

[B9] Casarin M. Todesco M. Sandrin D. Romanato F. Bagno A. Morlacco A. (2022c). A novel hybrid membrane for urinary conduit substitutes based on small intestinal submucosa coupled with two synthetic polymers. J. Funct. Biomater. 13, 222. 10.3390/jfb13040222 36412863 PMC9680483

[B10] Chemla E. S. Morsy M. (2009). Randomized clinical trial comparing decellularized bovine ureter with expanded polytetrafluoroethylene for vascular access. Br. J. Surg. 96, 34–39. 10.1002/bjs.6434 19108001

[B11] Cheng Y. Xiong X. Z. Zhou R. X. Deng Y. L. Jin Y. W. Lu J. (2016). Repair of a common bile duct defect with a decellularized ureteral graft. World J. Gastroenterol. 22, 10575–10583. 10.3748/wjg.v22.i48.10575 28082809 PMC5192268

[B12] Clarke D. R. Lust R. M. Sun Y. S. Black K. S. Ollerenshaw J. D. (2001). Transformation of nonvascular acellular tissue matrices into durable vascular conduits. Ann. Thorac. Surg. 71 (5 Suppl. l), S433–S436. 10.1016/s0003-4975(01)02556-5 11388242

[B13] Dahms S. E. Piechota H. J. Nunes L. Dahiya R. Lue T. F. Tanagho E. A. (1997). Free ureteral replacement in rats: regeneration of ureteral wall components in the acellular matrix graft. Urology 50 (5), 818–825. 10.1016/S0090-4295(97)00391-9 9372902 PMC7133180

[B14] Darby C. R. Roy D. Deardon D. Cornall A. (2006). Depopulated bovine ureteric xenograft for complex haemodialysis vascular access. Eur. J. Vasc. Endovascular Surg. 31, 181–186. 10.1016/j.ejvs.2005.07.006 16129632

[B15] Davis N. F. Cunnane E. M. O’Brien F. J. Mulvihill J. J. Walsh M. T. (2018). Tissue engineered extracellular matrices (ECMs) in urology: evolution and future directions. Surgeon 16, 55–65. 10.1016/j.surge.2017.07.002 28811169

[B16] de Jonge P. K. J. D. Simaioforidis V. Geutjes P. J. Oosterwijk E. Feitz W. F. J. (2015). Recent advances in ureteral tissue engineering. Curr. Urol. Rep. 16, 465–467. 10.1007/s11934-014-0465-7 25404179 PMC4234891

[B17] De Jonge P. K. J. D. Sloff M. Janke H. P. Versteegden L. R. M. Kortmann B. B. M. De Gier R. P. E. (2018). Ureteral reconstruction in goats using tissue-engineered templates and subcutaneous preimplantation. Tissue Eng. Part A 24, 863–872. 10.1089/ten.tea.2017.0347 29105596

[B18] Derham C. Yow H. Ingram J. Fisher J. Ingham E. Korrosis S. A. (2008). Tissue engineering small-diameter vascular grafts: preparation of a biocompatible porcine ureteric scaffold. Tissue Eng. Part A 14, 1871–1882. 10.1089/ten.tea.2007.0103 18950273

[B19] Emrecan B. Yilik L. Özbek C. Gürbüz A. (2006). Bovine ureter graft for haemodialysis access surgery. Nephrol. Dial. Transplant. 21, 2290–2291. 10.1093/ndt/gfl164 16632558

[B20] Field P. (2003). The chemically treated bovine ureter—clinical performance of a novel biological vascular prosthesis. Cardiovasc. Surg. 11, 30–34. 10.1016/s0967-2109(02)00113-8 12543569

[B21] Fontanella C. G. Toniolo I. Foletto M. Prevedello L. Carniel E. L. (2022). Mechanical behavior of subcutaneous and visceral abdominal adipose tissue in patients with obesity. Processes 10, 1798. 10.3390/pr10091798

[B22] Fröber R. (2007). Surgical anatomy of the ureter. BJU Int. 100, 949–965. 10.1111/j.1464-410X.2007.07207.x 17822477

[B23] Geavlete P. Georgescu D. Mulțescu R. Stanescu F. Cozma C. Geavlete B. (2021). Ureteral stent complications – experience on 50,000 procedures. J. Med. Life 14, 769–775. 10.25122/jml-2021-0352 35126746 PMC8811679

[B24] Hennicke N. S. Saemann M. Kluess D. Bader R. Sander M. (2022). Subject specific finite element modelling of periprosthetic femoral fractures in different load cases. J. Mech. Behav. Biomed. Mater 126, 105059. 10.1016/J.JMBBM.2021.105059 34995835

[B25] Hiroshi M. Hideaki K. Yuji N. Ken-Ichiro H. Yoshonari O. Shinichi O. (2006). Constructing a tissue-engineered ureter using a decellularized matrix with cultured uroepithelial cells and bone marrow-derived mononuclear cells. Tissue Eng. 12, 509–518. 10.1089/ten.2006.12.509 16579684

[B26] Janke H. P. de Jonge P. K. J. D. Feitz W. F. J. Oosterwijk E. (2019). Reconstruction strategies of the ureter and urinary diversion using tissue engineering approaches. Tissue Eng. Part B Rev. 25, 237–248. 10.1089/ten.teb.2018.0345 30794111

[B27] Ketharanathan V. Christie B. A. (1982). Bovine ureter as as vascular prosthesis: a preliminary report on an experimental study in dogs. Aust. N. Z. J. Surg. 52, 590–593. 10.1111/j.1445-2197.1982.tb06120.x 6819849

[B28] Kloskowski T. Kowalczyk T. Nowacki M. Drewa T. (2013). Tissue engineering and ureter regeneration: is it possible? Int. J. Artif. Organs 36, 392–405. 10.5301/ijao.5000130 23645581

[B29] Knight R. B. Hudak S. J. Morey A. F. (2013). Strategies for open reconstruction of upper ureteral strictures. Urologic Clin. N. Am. 40, 351–361. 10.1016/j.ucl.2013.04.005 23905933

[B30] Koch H. Hammer N. Ossmann S. Schierle K. Sack U. Hofmann J. (2015). Tissue engineering of ureteral grafts: preparation of biocompatible crosslinked ureteral scaffolds of porcine origin. Front. Bioeng. Biotechnol. 3, 89. 10.3389/fbioe.2015.00089 26157796 PMC4477215

[B31] Liu Y. Bharadwaj S. Lee S. J. Atala A. Zhang Y. (2009). Optimization of a natural collagen scaffold to aid cell-matrix penetration for urologic tissue engineering. Biomaterials 30, 3865–3873. 10.1016/j.biomaterials.2009.04.008 19427687

[B32] Mascolini M. V. Fontanella C. G. Berardo A. Carniel E. L. (2023). Influence of transurethral catheters on urine pressure-flow relationships in males: a computational fluid-dynamics study. Comput. Methods Programs Biomed. 238, 107594. 10.1016/J.CMPB.2023.107594 37207463

[B33] Matsuura J. H. Black K. S. Levitt A. B. Rosenthal D. Wellons E. D. Fallon M. T. (2004). Cellular remodeling of depopulated bovine ureter used as an arteriovenous graft in the canine model. J. Am. Coll. Surg. 198, 778–783. 10.1016/j.jamcollsurg.2004.01.020 15110812

[B34] Meng L. C. Liao W. B. Yang S. X. Xiong Y. H. Song C. Liu L. Q. (2015). Seeding homologous adipose-derived stem cells and bladder smooth muscle cells into bladder submucosa matrix for reconstructing the ureter in a rabbit model. Transpl. Proc. 47, 3002–3011. 10.1016/j.transproceed.2015.10.035 26707328

[B35] Murase Y. Narita Y. Kagami H. Miyamoto K. Ueda Y. Ueda M. (2006). Evaluation of compliance and stiffness of decellularized tissues as scaffolds for tissue-engineered small caliber vascular grafts using intravascular ultrasound. ASAIO J. 52, 450–455. 10.1097/01.mat.0000227727.87476.5e 16883127

[B36] Narita Y. Kagami H. Matsunuma H. Murase Y. Ueda M. Ueda Y. (2008). Decellularized ureter for tissue-engineered small-caliber vascular graft. J. Artif. Organs 11, 91–99. 10.1007/s10047-008-0407-6 18604613

[B37] O’Meara S. Cunnane E. M. Croghan S. M. Cunnane C. V. Walsh M. T. O’Brien F. J. (2024). Mechanical characteristics of the ureter and clinical implications. Nat. Rev. Urol. 21, 197–213. 10.1038/s41585-023-00831-1 38102385

[B38] Osman Y. Shokeir A. Gabr M. El-Tabey N. Mohsen T. El-Baz M. (2004). Canine ureteral replacement with long acellular matrix tube: is it clinically applicable? J. Urology 172, 1151–1154. 10.1097/01.ju.0000134886.44065.00 15311060

[B39] Pettenuzzo S. Belluzzi E. Pozzuoli A. Macchi V. Porzionato A. Boscolo-Berto R. (2023). Mechanical behaviour of plantar adipose tissue: from experimental tests to constitutive analysis. Bioeng. (Basel) 11, 42. 10.3390/bioengineering11010042 PMC10813593 38247919

[B40] Rassoli A. Shafigh M. Seddighi A. Seddighi A. Daneshparvar H. Fatouraee N. (2014). Biaxial mechanical properties of human ureter under tension. Urol. J. 11, 1678–1686. 10.22037/uj.v11i3.2472 25015616

[B41] Riva M. Hiepe P. Frommert M. Divenuto I. Gay L. Sciortino T. (2019). Intraoperative computed tomography and finite element modelling for multimodal image fusion in brain surgery. Oper. Neurosurg. 18, 531–541. 10.1093/ons/opz196 31342073

[B42] Roberts G. Mccormack H. Ketharanathan V. Macleish D. G. Field P. L. Milne P. Y. (1989). The role of physical and chemical characteristics in assessing the performance of a new biological vascular graft. J. Biomed. Mater Res. 23 (4), 443–450. 10.1002/jbm.820230405 2708417

[B43] Salmaso C. Toniolo I. Fontanella C. G. Da Roit P. Albanese A. Polese L. (2020). Computational tools for the reliability assessment and the engineering design of procedures and devices in bariatric surgery. Ann. Biomed. Eng. 48, 2466–2483. 10.1007/s10439-020-02542-9 32472365

[B44] Shalhav A. L. Elbahnasy A. M. Bercowsky E. Kovacs G. Brewer A. Maxwell K. L. (1999). Laparoscopic replacement of urinary tract segments using biodegradable materials in a large-animal model. Mary Ann. Liebert, Inc. 13, 241–244. 10.1089/end.1999.13.241 10405899

[B45] Shilo Y. Pichamuthu J. E. Averch T. D. Vorp D. A. (2014). First prize: evaluation of the tensile strength of the human ureter-preliminary results. J. Endourol. 28, 1470–1473. 10.1089/end.2014.0226 25343358

[B46] Sokolis D. P. (2019). *In vitro* study of age-related changes in human ureteral failure properties according to region, direction, and layer. Proc. Inst. Mech. Eng. H. 233, 570–583. 10.1177/0954411919839891 30922180

[B47] Spark J. I. Yeluri S. Derham C. Wong Y. T. Leitch D. (2008). Incomplete cellular depopulation may explain the high failure rate of bovine ureteric grafts. Br. J. Surg. 95, 582–585. 10.1002/bjs.6052 18344206

[B48] Steer J. W. Worsley P. R. Browne M. Dickinson A. (2021). Key considerations for finite element modelling of the residuum-prosthetic socket interface. Prosthet. Orthot. Int. 45, 138–146. 10.1177/0309364620967781 33176573

[B49] Thomas G. S. Gettman M. Lindberg G. Napper C. Pearle M. S. Cadeddu J. A. (2002). Ureteral replacement using porcine small intestine submucosa in A porcine model. Urology 60 (5), 931–934. 10.1016/s0090-4295(02)01890-3 12429340

[B50] Todesco M. Luisetto R. Casarin M. Imran S. J. Gerosa G. Fontanella C. G. (2023). “Biological evaluation *in vitro* and *in vivo* of hybrid membrane to assess biomedical application,” in Convegno Nazionale di Bioingegneria.

[B51] Tolva V. Bertoni G. B. Trimarchi S. Grassi V. Fusari M. Rampoldi V. (2007). Unreliability of depopulated bovine ureteric xenograft for infra inguinal bypass surgery: mid-term results from two vascular centres. Eur. J. Vasc. Endovascular Surg. 33, 214–216. 10.1016/j.ejvs.2006.10.007 17127082

[B52] Toniolo I. Fontanella C. G. Foletto M. Carniel E. L. (2022). Coupled experimental and computational approach to stomach biomechanics: towards a validated characterization of gastric tissues mechanical properties. J. Mech. Behav. Biomed. Mater 125, 104914. 10.1016/J.JMBBM.2021.104914 34715641

[B53] Toniolo I. Fontanella C. G. Gagner M. Stefanini C. Foletto M. Carniel E. L. (2021). Computational evaluation of laparoscopic sleeve gastrectomy. Updat. Surg. 73, 2253–2262. 10.1007/s13304-021-01046-y PMC8606391 33817769

[B54] Toniolo I. Pirini P. Perretta S. Carniel E. L. Berardo A. (2024). Endoscopic versus laparoscopic bariatric procedures: a computational biomechanical study through a patient-specific approach. Comput. Methods Programs Biomed. 243, 107889. 10.1016/j.cmpb.2023.107889 37944398

[B55] Van Mastrigt R. Glerum J. J. Tauecchio E. A. (1981). Variation of passive mechanical properties of the ureter along its length. Urol. Int. 36 (3), 145–151. 10.1159/000280405 7281372

[B56] Vardar E. Engelhardt E. M. Larsson H. M. Mouloungui E. Pinnagoda K. Hubbell J. A. (2015). Tubular compressed collagen scaffolds for ureteral tissue engineering in a flow bioreactor system. Tissue Eng. Part A 21, 2334–2345. 10.1089/ten.tea.2015.0048 26065873

[B57] Wang P. Xiao S. Fu W. Wang Z. Zhang X. (2021). A preliminary study on the promotion of canine adipose-derived stem cell differentiation by perfusion-decellularized ureter matrix. Transpl. Proc. 53, 2052–2059. 10.1016/j.transproceed.2021.06.008 34247859

[B58] Weiss R. M. Bassett A. L. Hoffman B. F. Hoff-B. F. (1972). Dynamic length-tension curves of cat ureter. Am. J. Physiol. 222 (2), 388–393. 10.1152/ajplegacy.1972.222.2.388 5058379

[B59] Wolters H. H. Heistermann H. P. Stppeler S. Hierlemann H. Spiegel H. U. Palmes D. (2010). A new technique for ureteral defect lesion reconstruction using an autologous vein graft and a biodegradable endoluminal stent. J. Urology 184, 1197–1203. 10.1016/j.juro.2010.04.072 20663520

[B60] Xiao S. W. Wang P. C. Fu W. J. Wang Z. X. Li G. Zhang X. (2016). Novel perfusion-decellularized method to prepare decellularized ureters for ureteral tissue-engineered repair. J. Biosci. Bioeng. 122, 758–764. 10.1016/j.jbiosc.2016.06.009 27405270

[B61] Xu Q. Chen C. Xu Z. Chen F. Yu Y. Hong X. (2020). Ureteral reconstruction with decellularized small intestinal submucosa matrix for ureteral stricture: a preliminary report of two cases. Asian J. Urol. 7, 51–55. 10.1016/j.ajur.2019.03.004 31970072 PMC6962721

[B62] Yin F. C. Fung Y. C. (1971). Mechanical properties of isolated mammalian ureteral segments. Am. J. Physiology-Legacy Content 221, 1484–1493. 10.1152/ajplegacy.1971.221.5.1484 5124294

[B63] Zhao Z. Liu D. Chen Y. Kong Q. Li D. Zhang Q. (2019). Ureter tissue engineering with vessel extracellular matrix and differentiated urine-derived stem cells. Acta Biomater. 88, 266–279. 10.1016/j.actbio.2019.01.072 30716556

